# Essential advances in surgical and adjuvant therapies for colorectal cancer 2018‐2019

**DOI:** 10.1002/ags3.12307

**Published:** 2020-01-28

**Authors:** Tomonori Akagi, Masafumi Inomata

**Affiliations:** ^1^ Department of Gastroenterological and Pediatric Surgery Faculty of Medicine Oita University Yufu‐City Japan

**Keywords:** adjuvant chemotherapy, colorectal cancer, surgical treatment

## Abstract

Surgical resection and adjuvant chemotherapy are the only treatment modalities for localized colorectal cancer that can obtain a “cure.” The goal in surgically treating primary colorectal cancer is complete tumor removal along with dissection of systematic D3 lymph nodes. Adjuvant treatment controls recurrence and improves the prognosis of patients after they undergo R0 resection. Various clinical studies have promoted the gradual spread and clinical use of new surgical approaches such as laparoscopic surgery, robotic surgery, and transanal total mesorectal excision (TaTME). Additionally, the significance of adjuvant chemotherapy has been established and it is now recommended in the JSCCR (the Japanese Society for Cancer of the Colon and Rectum) guideline as a standard treatment. Herein, we review and summarize current surgical treatment and adjuvant chemotherapy for localized colorectal cancer and discuss recent advances in personalized medicine related to adjuvant chemotherapy.

## Abstract 2

Patients with colorectal cancer considered to be curative now have several options for surgical approaches, adjuvant chemotherapy regimens, and duration of therapy. Although the current situation makes it difficult for oncologists and patients to make clinical decisions, future developments are expected to lead to more effective personalized medicines and treatment.

## 1 INTRODUCTION

According to the Vital Statistics of Japan, the number of deaths from colorectal cancer in Japan has continued to increase and, in 2016, it exceeded 50 000.^1^ The basis of surgical treatment for colorectal cancer has continued to be primary resection with lymph node dissection. However, new approaches such as sphincter preservation surgery, transanal total mesorectal excision (TaTME), robotic surgery, and laparoscopic surgery have been spreading. Important outcomes from the Japan Clinical Oncology Group (JCOG) 0404 trial were published indicating that laparoscopic surgery could be an acceptable option for patients with stage II or III colon cancer. Herein, we review and summarize the results of laparoscopic surgery and new approaches such as robotic surgery and TaTME.

The benefit of adjuvant chemotherapy has been confirmed in curatively resected stage III colon cancer, and it is now a standard treatment strategy in the guidelines of the Japanese Society for Cancer of the Colon and Rectum (JSCCR).^2^ The standard adjuvant chemotherapy regimen for colon cancer has been improved based on the findings from several large clinical trials. Ever since the significant benefit of adding oxaliplatin was proved,^3^, ^4^ creating other effective regimens has been difficult because several trials showed that no additional benefit was gained by adding bevacizumab or cetuximab.^5^, ^6^, ^7^ Thus, the prolonged neuropathy induced by oxaliplatin has emerged as a critical issue, and several prospective trials were conducted to test reductions in the duration of oxaliplatin treatment.^8^ A prospective, pre‐planned pooled analysis of six concurrently conducted randomized phase III trials (IDEA collaboration), including the ACHIEVE trial, was performed to evaluate the non‐inferiority of 3 vs 6 months of adjuvant FOLFOX/XELOX therapy. Although this study produced negative results, the authors suggested the possibility of adjusting the duration of adjuvant chemotherapy for stage III colon cancer according to the patient's risk and regimen and indicated the increasing importance of personalized medicine.

## 2 GENERAL PRINCIPLES OF RESECTION FOR COLORECTAL CANCER

Surgical resection of primary colon cancer is performed to completely remove the tumor, major vascular pedicles, and lymphatic drainage basin of the affected colonic and rectal segment and is achievable through an open or laparoscopic approach. However, the same principles of resection with lymph node dissection applicable to open surgery are also applicable to the laparoscopic approach.

Although the concept of complete mesocolic excision (CME) has begun to emerge in recent years,^9^ Japanese surgeons are already performing D3 lymph node dissection. Japanese D3 dissection and the current standard procedure of CME with central vascular ligation performed in the USA and Europe are almost identical although the resected colon is left slightly shorter following the Japanese D3 procedure. Theoretically, although the procedures should be equivalent because the principles are the same, a 2012 study^9^ showed CME with central vascular ligation and Japanese D3 dissection to be superior to the procedure used in previously reported cases.

Additionally, the Japanese specific lymph node dissection is pelvic lateral lymph node dissection, which is considered a distant metastasis in Western countries. Lateral lymph node dissection is indicated when the lower border of the tumor is located distal to the peritoneal reflection and the tumor has invaded beyond the muscularis propria. Prophylactic lateral lymph node dissection has a weak recommendation in the JSCCR guideline.^2^

## 3 GUIDELINES FOR COLORECTAL CANCER

The combination of advances in diagnostic methods and the use of many newly developed treatment methods has steadily improved the results of treatment. However, treatment methods vary among the medical institutions in Japan that treat patients with colorectal cancer, and this can lead to differences in treatment results.

Therefore, the JSCCR guidelines 2019 for the treatment of colorectal cancer,^2^ which are intended for surgeons and medical oncologists who provide medical care to patients with colorectal cancer of various stages and conditions, were prepared in order to (a) describe the standard treatment strategies for colorectal cancer, (b) eliminate treatment disparities among institutions, (c) eliminate unnecessary and insufficient treatments, and (d) deepen mutual understanding between healthcare professionals and patients by making these guidelines available to the general public.^1^

## 4 OPEN VS LAPAROSCOPIC COLECTOMY

Laparoscopic surgery, which is the standard treatment for colon cancer in the USA and Europe, has several benefits over open surgery in terms of short‐term outcomes such as decreased pain, improved postoperative pulmonary function, reduced postoperative ileus, lower incidence of wound infection, faster recovery, and shorter hospital stay. Further, as shown by the results of several randomized controlled trials,^10^, ^11^, ^12^, ^13^, ^14^, ^15^, ^16^, ^17^, ^18^, ^19^ the long‐term outcomes after laparoscopic surgery for colon cancer are comparable to those after open surgery. The key studies are summarized in Table 1. However, these trials were performed in the USA and Europe before the standard procedure of CME with central vascular ligation had emerged as a viable technique. These trials also had several weaknesses: the proportion of patients with tumors of pathological stage 0‐I was high (21%‐37%), some trials included rectal cancer, and the extent of lymph node dissection was not specified. To overcome these weaknesses in the previous trials, in the JCOG0404 trial the study population was limited to patients with clinical stage II or III colon cancer.^20^ Moreover, the protocol specified a detailed surgical procedure in which D3 dissection, which is similar to CME with central vascular ligation, was mandatory in all patients. This study was the first to compare and report long‐term outcomes following laparoscopic surgery vs open surgery requiring D3 dissection for clinical stage II or III colon cancer. Statistically, laparoscopic D3 surgery was not non‐inferior to open D3 surgery in terms of overall survival. However, possible reasons for failure of the present study statically were considered that overall survival in both groups was similar and better than expected, and the number of events observed — 50% of expected deaths had occurred — was insufficient. Laparoscopic D3 surgery was concluded to be an acceptable treatment option for patients with stage II or III colon cancer.

**Table 1:** Key RCTs of colon cancer

Authors	Journal	Cases	Conversion rate	5‐y overall survival (Open vs Lap)	5‐y disease‐free survival (Open vs Lap)
COST Ref. 12	N Eng J Med (2004)	869			
		Open 428	21%	85% vs 86%	81% vs 80%
		Lap 435		(*P* = .51)	(*P* = .50)
CLASSIC Ref. 16	Lancer (2005)	794			
		Open 268	16%	68% vs 67%	68% vs 66%
		Lap 525		(*P* = .35)	(*P* = .75)
COLOR Ref. 15	Lancer Oncol (2009)	1076			
		Open 542	17%	74% vs 74%	68% vs 67%
		Lap 534		(*P* = .40)	(*P* = 1.40)
JCOG0404 Ref. 20	Lancer GH (2017)	1057		90.4% vs 91.8%	79.7% vs 79.3%
		Open 524	5.40%	(*P* = .073)	(*P* = 1.40)
		Lap 524			

Abbreviation: RFS, Relapse‐free survival.

Several meta‐analyses of randomized trials also showed faster recovery and no detrimental impact on recurrence or survival rates for laparoscopic vs open colectomy for the treatment of colon cancer.^11^, ^12^, ^13^, ^14^, ^15^, ^17^, ^21^, ^22^, ^23^, ^24^, ^25^ Thus, if a surgeon with experience in advanced laparoscopic colectomy techniques is available, laparoscopic colectomy is now recommended for patients with uncomplicated localized colon cancers along with open surgery. Long‐term outcomes following laparoscopic vs open colon resection were addressed in two meta‐analyses of randomized trials, most of which exclusively or predominantly enrolled patients with colon cancer.^21^, ^22^ Results were analyzed from seven published randomized trials comprising 1536 patients in one meta‐analysis and four trials with an endpoint of survival that enrolled more than 150 patients in the other.^11^, ^12^, ^13^, ^26^ Both concluded that laparoscopic colectomy provides several oncologic outcomes, including number of lymph nodes harvested, disease recurrence, and overall survival (OS), that compare with those attained by the open approach. In the larger meta‐analysis, the rates of 3‐year disease‐free survival (DFS) for laparoscopic vs open colectomy were 75.8% and 75.3%, respectively (95% confidence interval [CI] of the difference, −5% to 4%), with similar rates of 3‐year OS of 82.2% vs 83.5% (95% CI, −3% to 5%).^21^ In the seven trials reporting this outcome, incisional recurrence occurred in only three of 826 patients randomized to laparoscopic surgery and in only one of the 801 patients who underwent open surgery.^22^

As another possible benefit of laparoscopic surgery, adjuvant chemotherapy is more likely to be started in patients who undergo laparoscopic colectomy for node‐positive cancer. Among 12 849 patients who underwent colectomy for stage III colon cancer, those undergoing the laparoscopic approach were more frequently administered adjuvant chemotherapy than those undergoing open colectomy (72% vs 67%).^27^ Because adjuvant chemotherapy is typically started only after recovery from surgery, laparoscopy might result in fewer complications and faster postoperative recovery.

## 5 OPEN VS LAPAROSCOPIC RECTAL RESECTION

Rectal cancer surgery is performed via the open, laparoscopic, and robotic approaches and is chosen based on surgeon and patient preference. No approach has been shown to be superior to another. Medical insurance coverage for the robotic approach began in Japan in 2019.

Traditionally, curative resection of rectal carcinoma was obtained by the open surgical techniques of low anterior resection or abdominoperineal resection. Laparoscopic rectal cancer surgery was compared with open surgery in four randomized trials that reported conflicting results. Therefore, until further data become available, the best surgical approach should be determined individually based on tumor and patient characteristics and surgeon experience. Among these trials, the European Colorectal cancer Laparoscopic or Open Resection (COLOR II) multicenter trial randomly assigned 1044 patients with a solitary rectal adenocarcinoma located within 15 cm of the anal verge to undergo laparoscopic or open surgery.^28^ The rates of macroscopic completeness of resection (88% vs 92%) and positive (<2 mm) circumferential resection margin (10% vs 10%) were similar between the two groups, as were the 3‐year rates of locoregional recurrence and survival.^29^ In the COREAN South Korean trial, 340 patients with mid‐to‐low rectal cancer were randomly assigned to undergo laparoscopic or open surgery after preoperative chemoradiation therapy.^30^ There were no significant differences in involvement of the circumferential resection margin, macroscopic quality of the total mesorectal excision specimen, number of harvested lymph nodes, or perioperative morbidity between the two groups. The 3‐year rates of DFS for the open and laparoscopic surgery groups were similar (72.5% vs 79.2%).^31^ In the ACOSOG Z6051 trial, which was designed to investigate non‐inferiority of the laparoscopic approach, 486 patients with stage II or III rectal cancer within 12 cm of the anal verge were randomly assigned to undergo laparoscopic or open surgery after neoadjuvant therapy.^32^ The primary endpoint was successful pathologic outcome, defined as simultaneous achievement of a >1‐mm distal margin, >1‐mm circumferential radial margin, and adequate total mesorectal excision. Successful outcomes were achieved by 81.7% of the patients undergoing laparoscopic resection vs 86.9% undergoing open resection. Laparoscopic surgery did not meet the non‐inferiority criteria. In a follow‐up study, comparable rates were reported for 2‐year DFS (laparoscopic 79.5% vs open 83.2%), local and regional recurrence (4.6% vs 4.5%), and distant recurrence (14.6% vs 16.7%).^33^ In another non‐inferiority trial, the Australian Laparoscopic Cancer of the Rectum Trial (AlaCaRT), 475 patients with T1 to T3 rectal cancer <15 cm from the anal verge were randomly assigned to laparoscopic or open resection.^34^ The primary endpoint was exactly the same as that in the ACOSOG Z6051 trial. Resection was successful in 82% of patients in the laparoscopic group vs 89% in the open surgery group, and again, laparoscopic surgery failed to meet the non‐inferiority criteria. Rates of 2‐year DFS reported in a follow‐up study were comparable (laparoscopic 80% vs open 82%), as were those for OS (94% vs 93%) and local and regional recurrence (5.4% vs 3.1%).^34^ These studies are summarized in Table 2.

**Table 2:** Key RCTs of rectal cancer

Authors	Journal	Cases	Primary endpoint	Conclusion
COREAN trial Ref. 30, 31	Lancet Oncol (2014)	869		
		Open 170	3 y‐DFS	Proved non‐inferiority of Lap
		Lap 170		
COLOR‐II trial Ref. 28, 29	N Eng J Med (2015)	794	3 y‐local recurrence free survival rate	Proved non‐inferiority of Lap
		Open 345		
		Lap 699		
ACOSOG‐Z6051 trial Ref. 32, 33	JAMA (2015)	462	Successful resection rate (CRM, DM, TME)	Not proved non‐inferiority of Lap
		Open 222		
		Lap 240		
ALaCaRT trial Ref. 34	JAMA (2015)	473	Successful resection rate (CRM, DM, TME)	Not proved non‐inferiority of Lap
		Open 235		
		Lap 238		

A systematic review and meta‐analysis of randomized trials that including the four above‐mentioned studies concluded that, for rectal cancer, a higher rate of “noncomplete” (composite of incomplete and near‐complete) total mesorectal excision was achieved with laparoscopic surgery than with open surgery (13.2% vs 10.4%). However, the rates of circumferential and distal margin involvement, mean numbers of lymph nodes retrieved, and mean distances to radial and distal margins were similar between the two techniques.^35^

## 6 ROBOTIC SURGERY

Robot‐assisted surgery is an emerging technology combining the advantages of the laparoscopic approach (e.g. faster recovery with less postoperative pain) with those of open surgery (e.g. high‐quality three‐dimensional view and restoration of the eye‐hand‐target axis).^36^, ^37^, ^38^, ^39^ As indicated by small retrospective reviews, in terms of lymph node harvesting and maintenance of negative radial margins, robot‐assisted total mesorectal excision is feasible, safe, and as efficacious as the open and laparoscopic approaches.^40^, ^41^, ^42^ Furthermore, three separate case‐matched analyses of patients undergoing robot‐assisted, laparoscopic, or open resection of mid‐ or low‐rectal cancers found no significant differences in oncologic outcomes.^43^, ^44^, ^45^ However, robot‐assisted surgery comes with the disadvantages of high cost and long set‐up and procedure times.^46^

Short‐term (6‐month) outcomes of 466 patients who underwent low anterior resection or abdominoperineal resection for rectal cancer were compared in the RObotic vs LAparoscopic Resection of Rectal Cancer Trial (ROLARR).^47^ Both robotic‐assisted and conventional laparoscopic surgery showed statistically similar rates of conversion to laparotomy (8.1% vs 12.2%), positive circumferential resection margin (5.1% vs 6.3%), mortality (0.9% vs 0.9%), overall complications (33.1% vs 31.7%), intraoperative complications (15.3% vs 14.8%), anastomotic leakage (12.2% vs 9.9%), and postoperative bladder or sexual dysfunction. Operative time with the robotic approach was longer by an average of 38 minutes, and hospital cost was higher by an average of £980 or US$1132 per patient.

The ROLARR trial showed that operative time was longer and cost was higher when robotic‐assisted laparoscopic rectal surgery was performed by surgeons with varying degrees of experience with robotic surgery, and it offered no incremental benefit over conventional laparoscopic surgery. Of note, the issue of oncologic equivalency of robotic‐assisted laparoscopic surgery to conventional or open rectal surgery was not addressed in the published data from the ROLARR trial.

A systematic review and meta‐analysis performed in 2018 of five trials, including ROLARR, comparing robotic‐assisted resection for rectal cancer with that of conventional laparoscopy found similar perioperative outcomes regarding mortality, rate of circumferential margin involvement, and number of lymph nodes harvested.^48^ Conversion from robotic surgery to open surgery was less likely (7.5% vs 12.9%), but robotic surgery took slightly longer than conventional laparoscopic surgery (mean difference 38 minutes).

## 7 TaTME

In centers with experienced surgeons, TME has also been attempted transanally for distal rectal tumors, particularly in obese men with a narrow pelvis.^49^, ^50^, ^51^ Because the distal margin can be assessed precisely from the beginning of the procedure, the resection margins can be defined more clearly in TaTME than in standard transabdominal TME. A randomized trial of patients with low rectal cancer (<6 cm from anal verge) that compared TaTME with standard TME showed a lower rate of positive circumferential resection margin for TaTME (4% vs 18%), with other outcomes being comparable between the groups.^52^

Other studies following up patients for up to 29 months showed comparable rates of local recurrence and survival between TaTME and standard TME.^50^, ^53^ However, long‐term oncologic outcomes of TaTME have not been reported yet. Additionally, iatrogenic urethral injury has been reported with TaTME in men.^54^ Presently, TaTME remains an investigational technique that should only be performed by experienced surgeons in high‐volume centers. For most patients with rectal cancer, transabdominal TME remains the standard treatment of choice.

## 8 ADJUVANT CHEMOTHERAPY FOR RESECTED STAGE III COLORECTAL CANCER

Among patients who have undergone a potentially curative resection, disease recurrence is thought to arise from clinically occult micrometastases present at surgery. The goal with postoperative (adjuvant) therapy is eradication of these micrometastases to increase the cure rate. The benefits of adjuvant chemotherapy have been most clearly demonstrated in stage III disease. The JSCCR guideline recommends several agents and regimens described below.

An oxaliplatin‐based regimen, rather than a fluoropyrimidine‐based regimen alone, is recommended for patients <70 years old with resected stage III disease who are expected to tolerate oxaliplatin. Multiple randomized trials have shown a survival benefit by adding oxaliplatin to adjuvant fluoropyrimidines in patients with resected stage III colon cancer, and the benefit appears across diverse practice settings and patient subgroups. However, the benefits of an oxaliplatin‐ over a non‐oxaliplatin‐containing regimen are less clear in patients ≥70 years old. In contrast, orally active fluoropyrimidines such as capecitabine and UFT are more convenient and may offer an improved therapeutic ratio.^55^ The benefit offered by oral fluoropyrimidines was investigated in a meta‐analysis of individual data from five Japanese trials comprising 5232 patients with resected stage I, II, or III colon cancer who were randomly assigned to receive adjuvant oral fluoropyrimidines (FU, UFT, or hexacarbonyl FU) or undergo observation only.^56^ Overall, oral therapy reduced the risk of recurrence by 11% and death by 15%. However, an absolute survival benefit of only 2.5% was achieved for patients with stage III disease.

More recent trials suggest that the benefit achievable with either capecitabine or UFT is at least equivalent to that of FU/LV administered by intravenous bolus. Two randomized trials of oral capecitabine compared with intravenous fluoropyrimidines showed equivalent rates of 6‐month DFS.^57^, ^58^ In the earlier trial, the European/Canadian Xeloda in Adjuvant Colon Cancer Therapy (X‐ACT) study, 1987 patients with resected stage III colon cancer were randomly assigned to 6 months of capecitabine alone.^57^ The trial was statistically powered to show therapeutic equivalence, and DFS was the primary endpoint.

Six months of UFT plus LV is a standard approach for adjuvant chemotherapy of stage III colon cancer in Japan.^35^, ^59^, ^60^ The NSABP C‐06 trial showed the comparative efficacy of UFT plus LV compared with parenteral FU/LV in non‐Asian populations comprising 1608 patients with resected stage II or III colon cancer who were randomly assigned to a weekly bolus of FU with high‐dose LV.^61^ The 5‐year rates of DFS or OS were not significantly different. A similar conclusion was reached in the JCOG0205 phase III trial of patients with stage III disease only.^60^ In a Japanese trial of 1535 patients with resected stage III colon cancer, the utility of S‐1 for adjuvant treatment of the cancer was determined by directly comparing S1 with UFT plus LV.^62^ S‐1 was found to be non‐inferior to UFT + LV (hazard ratio [HR] for DFS, 0.85; 95% CI, 0.70‐1.03), and the rates of adverse events were comparable. However, S‐1 was found to be inferior to capecitabine monotherapy in the JCOG0910 non‐inferiority multicenter randomized trial.^63^ At present, we should make clinical decision about adjuvant chemotherapy taking into account patient characteristics, values, and preferences, and the potential for benefit and risks of adverse events associated with treatment.

## 9 DURATION OF ADJUVANT CHEMOTHERAPY

The optimal duration of adjuvant oxaliplatin chemotherapy for patients with stage III colon cancer is still evolving. An analysis of the International Duration Evaluation of Adjuvant Chemotherapy (IDEA) collaboration (six separate randomized trials of 6 vs 3 months of adjuvant oxaliplatin‐based therapy) revealed that in the overall population of patients with stage III colon cancer receiving adjuvant therapy with FOLFOX or CAPOX, non‐inferiority of 3 vs 6 months of therapy was not confirmed. For the patients treated with CAPOX, and particularly those in the lower‐risk subgroup, 3 months was as effective as 6 months of therapy.

In a combined analysis of 12 834 patients with resected stage III colon cancer enrolled in all six trials, 3 months of oxaliplatin‐based therapy did not meet the prespecified criteria for non‐inferiority, as the 3‐year rate of DFS of patients with 3 months of therapy was 74.6% vs 75.5% for 6 months of therapy (HR, 1.07; 95% CI, 1.00‐1.15).^8^ However, 6 months of therapy was superior for the patients with high‐risk cancers (T4N2) as they experienced an absolute increase in 3‐year DFS of 1.7% (HR, 1.12; 95% CI, 1.03‐1.23). In the patients with low‐risk cancers (T1‐3N1), rates of 3‐year DFS were 83.1% and 83.3% for 3 and 6 months of treatment, respectively, which met the criteria for non‐inferiority (HR, 1.01; 95% CI, 0.90‐1.12). In a pre‐planned subgroup analysis, non‐inferiority of 3 vs 6 months of therapy was shown for patients receiving the CAPOX regimen (40% of analyzed patients; 3‐ vs 6‐month HR for recurrence, 0.95; 95% CI, 0.85‐1.06) but not FOLFOX (HR, 1.16; 95% CI, 1.06‐1.26). The ACHIVE study in Japan was one of the six prospective IDEA Collaboration trials and the only trial conducted in Asia. The ACHIVE study demonstrated that there were no differences in terms of efficacy among the other trials.^64^ The risk of adverse events was significantly reduced in those treated with 3 vs 6 months of therapy. Most importantly, the risk of grade 2 or higher neuropathy was markedly reduced in the 3‐month group (16.6% FOLFOX, 14.2% CAPOX) vs the 6‐month group (47.7% FOLFOX, 44.9% CAPOX).

The 2019 Clinical Practice Guideline from the American Society of Clinical Oncology (ASCO) states that patients with stage III colon cancer who are at a higher risk of recurrence (T4 and/or N2) should undergo 6 months of adjuvant oxaliplatin‐based chemotherapy, whereas lower‐risk patients can undergo either 3 or 6 months of therapy.^65^ The ASCO expert panel recommended a shared decision‐making approach that takes patient characteristics, values, and preferences, and the potential for benefit and risks of harm associated with treatment duration into account. No particular oxaliplatin‐containing regimen was recommended by the guideline for patients who undergo 3 months of adjuvant therapy, but they noted that 3 months of treatment was inferior to 6 months in the patients receiving FOLFOX but not CAPOX.

Six months of oxaliplatin‐based chemotherapy has been the standard of care for adjuvant treatment of stage III colon cancer. However, due to the cumulative and dose‐limiting peripheral neuropathy caused by oxaliplatin (grade 3 neuropathy in 13% of patients receiving 6 months of FOLFOX in the MOSAIC trial^66^), a shorter period of therapy might be advantageous if it is equally effective.

In Japan, the JSCCR has developed a nomogram to calculate risk of recurrence after curative resection that includes data on age, gender, tumor location, stage, and preoperative tumor markers from 19 institutions. This is expected to be an important information tool for use in personalized medicine. Additionally, a report based on data from NSABP C‐07 suggested that use of the Oncotype DX colon cancer assay for genomic profiling might improve risk prognostication in stage III disease and better clarify the absolute benefit that might be achieved by adding oxaliplatin to the therapy of these patients.^67^ High Recurrence Score values indicated a higher absolute benefit from oxaliplatin. However, even among the patients with the highest risk, stage IIIC disease, the absolute difference in the risk of recurrence at 5 years by adding oxaliplatin was small in the patients with a low Recurrence Score (FU treated: 41%; 95% CI, 28%‐57%, vs oxaliplatin treated: 38%; 95% CI, 23%‐58%) vs those with a high Recurrence Score (FU treated: 67%; 95% CI, 52%‐82% vs oxaliplatin treated: 59%; 95% CI, 42%–76%). Furthermore, the estimates are quite imprecise and may not be clinically meaningful. In recent years, the 12‐gene Oncotype DX assay, which is now available to Japanese patients,^68^ might allow the choice of agent and regimen according to the risk of recurrence. At present, we should make clinical decisions about adjuvant chemotherapy by taking into account patient characteristics, values, and preferences, and the potential for benefit and risks of adverse events associated with treatment.

## 10 CONCLUSION

Clinical outcomes of patients with colorectal cancer have improved as a result of the variety of surgical approaches and agents developed in this past decade. Patients with colorectal cancer considered to be curative now have several options for surgical approaches, adjuvant chemotherapy regimens, and duration of therapy. Although the current situation makes it difficult for oncologists and patients to make clinical decisions, future developments are expected to lead to more effective personalized medicines and treatment.

## DISCLOSURE

Conflicts of Interest: Authors declare no conflict of interest for this article.
